# Automated rapidplan model validation using Eclipse scripting API

**DOI:** 10.1002/acm2.70120

**Published:** 2025-05-11

**Authors:** Bradley Beeksma, Andrew Dipuglia, Joerg Lehmann

**Affiliations:** ^1^ Department of Radiation Oncology Calvary Mater Newcastle Warratah New South Wales Australia; ^2^ School of Information and Physical Sciences University of Newcastle Newcastle New South Wales Australia; ^3^ Institute of Medical Physics University of Sydney Sydney New South Wales Australia

**Keywords:** ESAPI, radiotherapy treatment planning automation, RapidPlan, scripting

## Abstract

RapidPlan offers efficiency gains and quality improvements in treatment planning. Prior to its use in the clinic, it requires an extensive validation procedure in which established clinical plans and those generated by the model are compared. The manual iterative nature of this process is resource intensive, as numerous iterations are required to fine‐tune and optimize a RapidPlan model. To streamline the efficiency and reduce the resource burden of RapidPlan model validation, a standalone executable auto planning script was written in C# leveraging the Eclipse scripting application programming interface (ESAPI). The script automatically batch generates treatment plans, as well as exports and plots the population‐based DVH metrics of these plans, without any user input, reducing the time and effort required to explore and refine model objectives. Configured with adjustable parameters via Excel and .txt files, this approach allows end users to change input variables quickly and easily without needing to re‐approve the script. The script has been implemented for a variety of treatment sites, including intact prostate, prostate & nodes, lung, rectum, unilateral head & neck, bilateral head & neck, and liver stereotactic body radiation therapy (SBRT). The process for liver SBRT has been used here as an example to illustrate the use and power of the script. Over numerous iterations, 76 patients in the model set and 17 patients in a validation set were replanned using the script, creating a total of 405 automatic plans with an overall active planning time of 118.7 h. This study demonstrates the effectiveness of automating the RapidPlan model validation process, significantly reducing the time and resource burden associated with traditional manual methods.

## 1 INTRODUCTION

RapidPlan (Varian Medical Systems, Palo, Alto, CA, USA) is a knowledge‐based planning (KBP) tool which assists in improving quality, consistency and efficiency of radiotherapy treatment plans.^1^ Utilizing a training set of acceptable relevant treatment plans, RapidPlan extracts patient anatomical geometric information and plan dosimetric information to train an individual organ‐at‐risk (OAR) dose volume histogram (DVH) estimation model. Using the patient structure set, dose prescription and beam geometry, RapidPlan is able to estimate OAR DVHs and generate optimization objectives aimed at achieving the estimated DVH range. This serves as a starting point for plan generation, thus improving efficiency and constancy in the planning process.^1^

While RapidPlan offers efficiency gains and quality improvements, its implementation necessitates an extensive validation process before clinical use. This involves detailed comparative analyses of dosimetric quality between established clinical plans and those generated by the model. This process is repeated across multiple different patient datasets that form a representative sample of the type of plans the model is intended for.^2^ As reported by Hussein et al.,^3^ an iterative process is required to fine‐tune and optimize a RapidPlan model. Only after numerous iterations of model refinement can a RapidPlan model produce plans of acceptable quality. Consequently, the process of validating such a model can be significantly time‐consuming and resource‐intensive due to the necessity of re‐planning across the entire validation set following any modifications to the DVH estimation models or optimization objectives.^4^ For each permutation of model version (M) and for each treatment plan in the validation set (*N*), the physicist or dosimetrist compares dosimetric plan quality of the RapidPlan‐generated plans to both the clinical plan and to plans generated by previous model versions. Based on this evaluation, changes to the model are either accepted or rejected. This process repeats until the model produces plans of acceptable plan quality across the entire validation set. Manually performing this validation loop is time consuming and resource intensive. Numerous model iterations and a large validation set quickly generate large amounts of data (MxN plans). Effectively extracting data and drawing conclusions from such large dataset can be difficult, laborious, and prone to errors. Investigating small model adjustments may become unviable from a resource perspective, and thus, end users may settle for an inferior model version where a more optimal solution may exist.

The Eclipse (Varian Medical Systems, Palo Alto, CA, USA) treatment planning system (TPS) allows developers to write custom software to interact with patient treatment plans. This is achieved through the Eclipse scripting application programming interface (ESAPI), which is a Microsoft .NET class library. ESAPI allows developers to create scripts that leverage the functionality of Eclipse to retrieve plan, image, dose, structure set, and DVH information from the Varian System database.^5^ From version 15.5 of Eclipse, ESAPI has write access to treatment plans, allowing developers to externally modify patient data via scripts. Not only can these scripts be called from the context of a patient plan, but ESAPI also permits standalone executables to modify patient data outside the context of the planning systems interface.^6^ This allows for batch planning software, which can iteratively run across multiple patient datasets.

To streamline the efficiency and reduce the resource burden of validating RapidPlan models, leveraging ESAPI, a standalone executable auto planning script was written in C#. The script automatically generates treatment plans for a specified number of patients, as well as exports and plots the population based DVH metrics of these plans without user input. This work presents a streamlined, efficient method for automating RapidPlan model validation with minimal user interaction.

## 2 METHODS

ESAPI requires the approval of any write‐enabled script before it can be run on the production server. This approval is enforced through ESAPI's strict version control, where any change to the scripts assembly version requires re‐approval.^7^ While this process is designed to facilitate the safe deployment of write‐enabled scripts, it prevents minor code variations or changes to variables in the code, as developers would continually be required to edit and approve scripts before running the next iteration. To address this challenge, the auto planning script was configured to utilize an Excel spreadsheet and .txt files as configuration files to read variable input data. This allowed end users to change input variables quickly and easily without needing to re‐approve the script. It also broadened the script's user base, as no programming knowledge is required to modify and run it. The configuration files encompass various variables, including patient medical record numbers (MRNs) and corresponding target dose levels, RapidPlan model ID, RapidPlan model structures, auto‐plan and course ID, reference plan ID, settings for normal tissue optimization, plan normalization, and DVH parameters for export and comparative analysis.

When the auto planning script is run, input variables from the aforementioned configuration files are loaded into the software and the following steps executed. Verification procedures embedded in the script determine if the validity of the input parameters is functional. This includes confirming the existence of a valid RapidPlan model and ensuring the uniqueness of the course and plan ID. In the event of a non‐unique course or plan ID, a trailing number is appended and incremented until a unique solution is found. Following validation of the configuration file parameters, the first patient plan is loaded and a new plan is created into the course variable as specified in the configuration file. Image set, structure set, prescription dose, and beam geometry from the reference plan are duplicated and applied to the new plan. All optimization and evaluation structures required for the RapidPlan model are automatically generated and the RapidPlan model version specified in the configuration files is applied. Dose values for each target structure for a given patient plan are added according to the data provided by the user in the Excel configuration file. This approach allows for planning at any specified dose level. Leveraging RapidPlan, the DVH estimation model is used to calculate DVH estimates which then automatically generate the plan optimization objective values. Once these are determined, the plan is optimized and the dose distribution is calculated using the system default optimization and dose calculation algorithms (PO and AcurosXB v15.6.05). If specified in the configuration files, the plan is then normalized and DVH metrics are exported to a .CSV file for both the automatically generated plan and the reference plan. These steps are then looped and performed for all subsequent patient MRNs specified in the excel configuration file.

**FIGURE 1:**
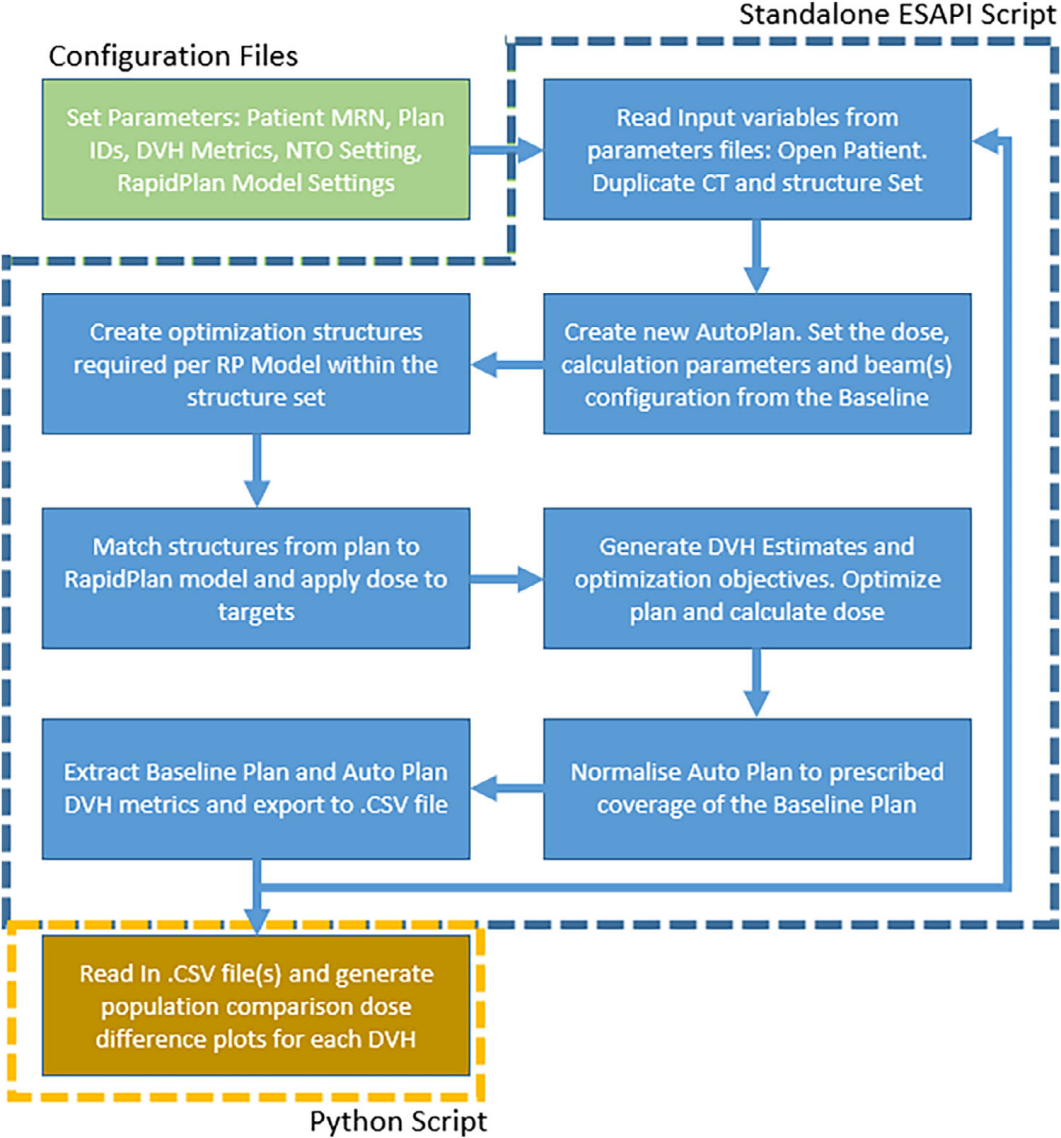
Flowchart of the actions performed by the automatic planning script.

Upon completion of the ESAPI script execution, population‐based DVH comparison metrics are automatically plotted using the estimation statistics tool,^8^ which has been implemented through a Python script. This process is executed with no user intervention required. Additionally, detailed log files are generated to document all interactions performed by the script, providing comprehensive debugging and progress reporting capabilities. A description of the auto planning script process is summarized in Figure 1.

The automatic planning script has been configured to automatically produce treatment plans, without manual interventions, for a variety of treatment sites, including intact prostate, prostate & nodes, lung, rectum, unilateral head & neck, bilateral head & neck, and liver stereotactic body radiation therapy (SBRT). The process for liver SBRT has been used here as an example to illustrate the use and power of the script.

Datasets from 76 patients previously treated at our institution for Liver SBRT were selected as training plans to build a Liver SBRT RapidPlan model. These plans are referred to throughout this paper as the model set. These plans had been manually planned to local departmental protocols based on the RTOG1112^9^ and CORE^10^ clinical trial protocols. All plans utilized a volumetric modulated arc therapy (VMAT) technique of two partial arcs and a 10 MV flattening filter free beam. Mean dose to the healthy liver was placed as the highest planning priority. In instances where achievable mean liver dose was not met, the prescription dose was lowered until the constraint was met. This resulted in prescriptions ranging from 60 to 24 Gy utilizing both three and five fraction regimens.

A further 17 liver SBRT patients plans were selected for model validation. This cohort of patients was not included within the model set. Having these plans independent of the model set allows testing the capability of the model to predict the planning outcome of patients unknown to the model.^11^ This mimics the clinical setting where the patient geometry is not contained within the RapidPlan trained data. The 17 validation plans that were exported, anonymized, and re‐imported into the TPS are referred to throughout this paper as the validation set. Although anonymization is not strictly necessary, since a script was being used to modify the patient data without user input, this step was performed to limit excessive data being generated in clinical patients, as well as a safety measure to eliminate the possibility of unintentionally corrupting clinical patient data.

The aim of the validation process was to determine the optimal model objectives to produce plans of an equivalent or better plan quality to the clinically accepted reference plan and to best meet the dose objectives specified by the planning protocols. Since this cyclic process is typically performed manually, time stamps from the log files were analyzed to quantify the efficiency gains performed by the script. Log files were assessed to establish the number of errors encountered by the script to determine its robustness to run without interruption.

## 3 RESULTS

Treatment plans were successfully batch produced automatically without user intervention, radically streamlining the efficiency of the RapidPlan validation process. As shown in Table 1, the 76 patients within the model set were re‐planned with M = 2 iterations to improve plan quality and consistency. During the validation loop, 17 cases were planned with M = 17 different model iterations before the optimal model objectives were achieved.

**TABLE 1:** Statistics for script‐generated automated plans.

	Model set	Validation set	Total
Total # patients plans	76	17	93
Auto replan—Fails	18	0	18
Auto replan—success (N)	58	17	75
Number of iterations (M)	2	17	19
Total # auto plans (MxN)	116	289	405
Total active planning time (hrs)	33.6	85.1	118.7

**FIGURE 2:**
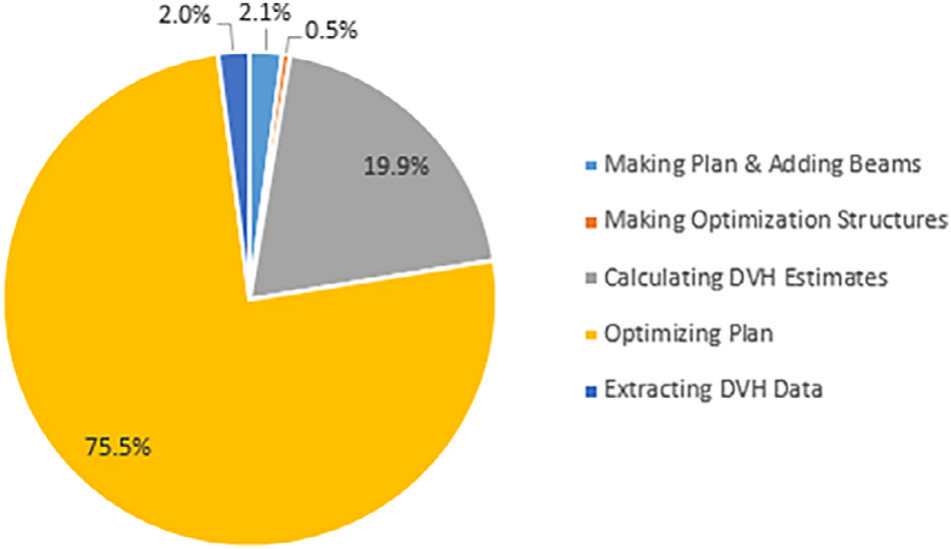
Average time script takes to perform each relative task per plan.

For the model set, the script was unable to create plans for 18 out of the 76 patients, leaving 58 successful plans generated per iteration. The script successfully generated plans for all patients in the validation set without failure. In total, 405 plans were automatically generated with an overall active planning time of 118.7 h, or on average, 17.3 min per plan. Figure 2 breaks down the average time per task that the script performs to generate an auto plan.

**FIGURE 3:**
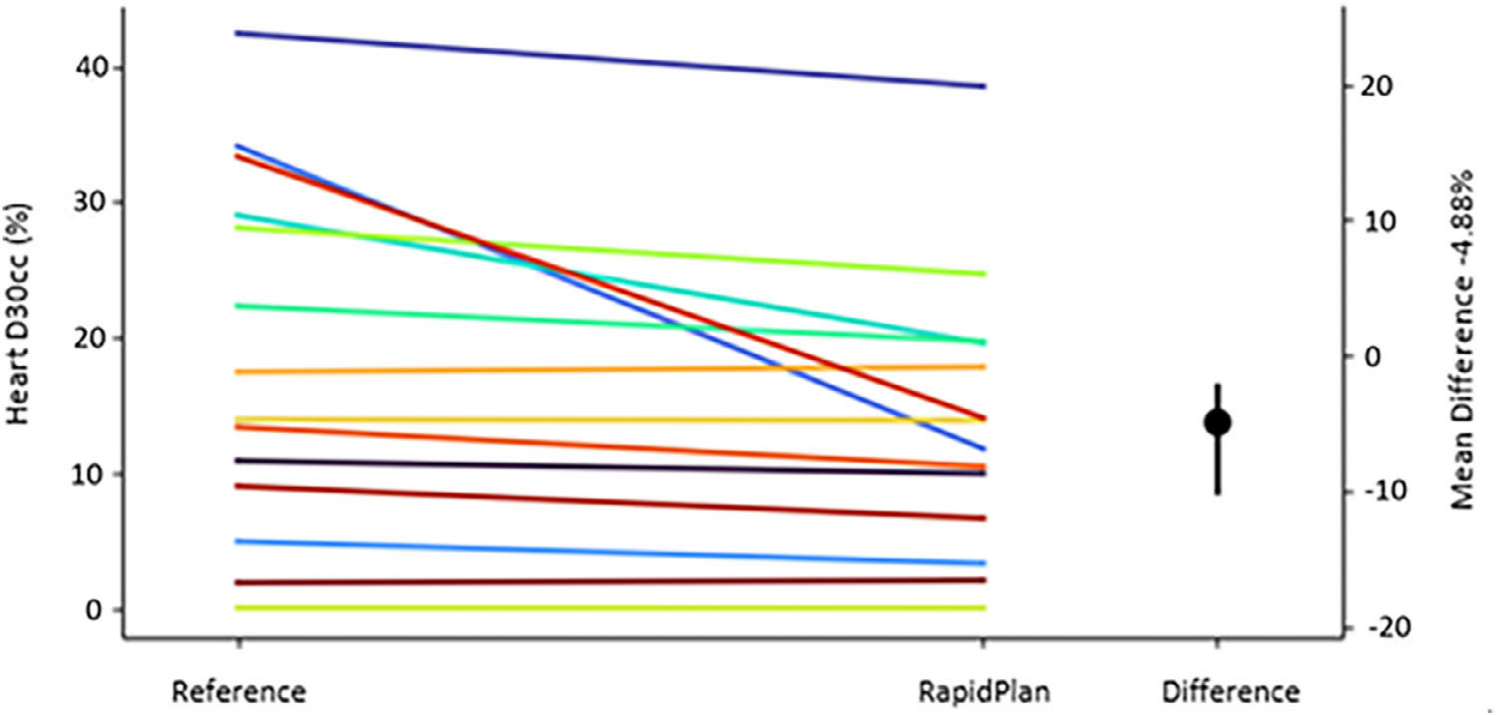
An example of population plots, which enables users to visually compare results for all automatic plans against the reference plans simultaneously for any given metric. Each coloured line represents one plan, drawing a connection between dose for the reference (clinical plan) and that of the automatically generated RapidPlan. The secondary plot axis illustrates the population mean difference (%) as a point, where the positive and negative error bars indicate the population min/max difference values.

Each time the script was executed, plots of various plan comparison metrics were automatically produced to assist with quantifying the difference between the automated and reference plans. Such metrics were customised and established from within the DVH configuration file. Population plots metrics included target coverage, target conformity, target homogeneity, OAR dose, monitor units, and modulation indices. An example plot for the validation set is shown in Figure 3 for the Heart D30cc. Lines with a negative slope indicate a decrease in the given metric between the reference plan and the auto plan, where a positive gradient indicates an increase. Utilization of these plots simplified large amounts of data into a single figure to assist with determining optimal model objectives, as any given metric for all plans can be visually viewed quickly and simultaneously. In addition to the visual representation of the data, the script produces a .CSV file which is automatically imported into an Excel spreadsheet using a custom macro written in visual basic analysis script. Once imported, the spreadsheet quantitatively evaluates the mean value of each DVH metric for the two datasets and additionally provides traffic light conditional formatting based on pass/fail criteria formulated from clinical objectives. The results presented in Table 2 depicts the average dosimetric difference between the reference and automatic plans, computed across the 17 plans in the validation set. The comparison was made after the final 17th iteration of the script. The analysis also includes a comparison with the clinical goal for each structure.

**TABLE 2:** Reference plan versus automatic plan dose metrics averaged across all validation cases.

Structure	Metric	Goal	Reference plan	Automatic plan
GTV_High	D100%	>100%	102.0 %	100.8 %
	D50%	>120%	109.6 %	120.9 %
	D2%	>125%, < 140%	113.5 %	132.6 %
PTV_High	D95%	>100%	100.0 %	100.0
Liver‐GTV_High	Mean	ALARA	8.66 Gy	8.75 Gy
Oesophagus	D0.1cc	<33 Gy	14.31 Gy	12.81 Gy
Stomach	D0.1cc	<32 Gy	11.19 Gy	11.15 Gy
	D10cc	<25 Gy	8.13 Gy	7.93 Gy
Duodenum	D0.1cc	<32 Gy	15.41 Gy	14.37 Gy
	D5cc	<25 Gy	9.92 Gy	8.35 Gy
Small bowel	D0.1cc	<32 Gy	8.49 Gy	6.80 Gy
	D10cc	<25 Gy	5.13 Gy	3.50 Gy
Large bowel	D0.1cc	<34 Gy	20.87 Gy	20.54 Gy
Heart	D0.1cc	<34 Gy	19.78 Gy	16.87 Gy
	D30cc	<30 Gy	7.80 Gy	6.51 Gy
Spinalcord PRV	D0.035cc	<25 Gy	6.54 Gy	7.08 Gy
Common bile duct	D0.1cc	<50 Gy	3.56 Gy	3.01 Gy
Gall bladder	D0.1cc	<55 Gy	20.60 Gy	18.06 Gy
Chest wall	D0.5cc	<50 Gy	28.70 Gy	28.17 Gy
MU/cGy		<4	3.13	3.51

*Note*: Data is given for the final version of the RapidPlan model achieved after the 17th and final iteration of the script.

## 4 DISCUSSION

The feasibility of using KBP to improve OAR sparing is well documented within the literature. Stanton et al.^12^ performed five iterations of re‐planning 40 breast plans contained within their model set. After each model iteration, they validated their model using 20 plans within their validation set. This equates to 300 replans performed manually. Fogliata et al.^13^ performed two iterations of re‐planning 83 Head and Neck plans within their model set, with a further three iterations of the 20 Plans in the validation set, equating to a total of 226 replans. Wang et al.^14^ performed three iterations of re‐planning 81 rectal cancer plan to the plans contained within the model set. Per model iteration, 30 plans were generated on the validation set, equating to 333 replans. Hence, model validation planning currently relies heavily on manual processes.

The aforementioned studies largely emphasize the dosimetric advantages and efficiency of RapidPlan, yet there is limited focus on the resources required for the development and validation of a model capable of producing clinically viable treatment plans. As highlighted by Meyer et al.^15^ while the primary strength of RapidPlan lies in its ability to generate DVH estimation curves and subsequent optimal planning objectives, its major limitation is the time‐intensive process of tuning model objectives. With the increasing availability of plan automation capabilities across commercial systems, there has been a substantial rise in studies aimed at reducing the time and effort required for plan generation through automation in treatment planning.^15^, ^16^ However, these studies often focus on case‐by‐case implementations and still frequently necessitate manual patient loading within the vendor‐specific software. Few investigations have explored the use of software to facilitate automated batch plan generation.

Li et al.^4^ created an ESAPI script to automate the re‐planning of 204 cervical cancer cases without the need for manual intervention, as part of a KBP‐driven quality control system for clinical trials. While their approach was effective, the authors provided limited detail on the software development process, focusing primarily on the dosimetric outcomes of the model rather than the specifics of its implementation. Harms et al.^17^ developed a .NET‐based application, RapidCompare, to automatically generate 75 lung cancer plans with limited manual intervention. The authors present a novel method to streamline the deployment of KBP models and provide some specifics of the user configurable software infrastructure. Both these studies were successfully able to automate the planning process but are limited to a single treatment site, do not give active timing data to quantify efficiency gains via the use of automation, nor discuss instances where the automatic planning software may fail to produce a plan.

The primary advantage of incorporating automation into the validation process is the capacity to rapidly generate and analyze substantial amounts of data with high efficiency. Utilizing ESAPI, this study demonstrates the successful development of software that enables users without coding expertise to execute batch planning. This approach was designed to evaluate the performance of a RapidPlan model, allowing for the automated testing of different optimization objectives without the need for time‐intensive manual replanning. As shown in Table 1, 17 different iterations of optimization parameters were tested to validate the model, considerably exceeding the number typically found in reported manual RapidPlan validation studies. This anecdotally suggests that the efficiency gains provided by the script allows the end user a tool to fine tune models, rather than settling for a perhaps a suboptimal endpoint.

Figure 2 shows that 95.4% of the total runtime was consumed by computational processes, including DVH estimation, optimization, and dose calculation. Eclipse optimization algorithms in v15.6.05 do not support GPU‐based VMAT optimization and, hence, the CPU was used here. Although GPU was utilized for dose calculation, distributed calculation framework (DCF) was not enabled, slowing the calculation process. Starting from Eclipse version 16, GPU optimization is supported by the PO algorithm. As most time in this study was spent on calculation and optimization, upgrading to a version with VMAT GPU optimization and using DCF is expected to significantly reduce the runtime for batch planning.

The script failed to generate a plan 18 out of 76 cases in the model set, but succeeded for all cases for all iterations in the validation set. Since the validation set was an anonymized dataset, it was “cleaned” prior to validation, meaning that structure names were manually changed to match the required input for the script perfectly. Conversely, reference plans in the model set were approved clinically treated plans, therefore were unable to be modified or “cleaned” to suit the input requirements of the script. The most common issue associated with the script failing to generate a plan was the script being unable to match structures to the RapidPlan model due to non‐compliant structure names. This reinforces the importance of naming standardization being a vital precursor to the development of scalable uses of scripting for quality assurance and treatment plan evaluation.^18^ Issues encountered less frequently were associated with the image set, plans referencing decommissioned dose calculation algorithms, or relative electron density curves in the TPS.

As shown in Table 2, a Liver SBRT RapidPlan model that satisfied the requirements of the clinical goals for all structures has been created. The automatically produced plans achieved superior dose sparing for 13 out of the 15 organ at risk metrics analyzed. Negligible differences in doses were seen in the two structures in which the reference plans achieved superior sparing, as the doses were well below the clinical goal. For target coverage, the reference plans failed to meet two clinical goals under covering the GTV_High D50% and D2%. Conversely, the automatic plans met all target coverage metric clinical goals. The trade‐off for sparing OAR can be seen by the modulation index (MI). Defined as the number of monitor units per Centigray, the reference plan has lower MI, indicating lower dose modulation compared to the automatic plans.

## 5 CONCLUSION

This study demonstrates the effectiveness of automating the RapidPlan model validation process, significantly reducing the time and resource burden associated with traditional manual methods. The developed automated method allows for rapid iteration and testing of multiple model configurations. While the script encountered minor issues with non‐standardized datasets, it performed flawlessly in the validation set, emphasizing the importance of data standardization in scaling automation tools for clinical use. Additionally, integrating GPU‐based optimization in future versions could further enhance the speed and efficiency of batch planning.

## AUTHOR CONTRIBUTIONS

**Bradley Beeksma**: had the original idea for the works, designed software infrastructure, and code development. Performed all data acquisition as well as interpretation and analysis of results. Primarily wrote the manuscript. **Andrew Dipuglia**: software design, code development, and contributed to drafting and editing of manuscript. **Joerg Lehmann**: concept discussions, drafting, and editing of manuscript. All authors reviewed and approved the final manuscript, agreeing to be accountable for the work's accuracy and integrity.

## CONFLICT OF INTEREST STATEMENT

The authors declare no conflicts of interest.
